# A Case of Renal Metastasis of Uterine Leiomyosarcoma

**DOI:** 10.7759/cureus.1470

**Published:** 2017-07-14

**Authors:** Petar Bajic, William S Gange, Robert H Blackwell, Arpeet S Shah, John Biemer, Maria M Picken, Alex Gorbonos

**Affiliations:** 1 Department of Urology, Loyola University Medical Center; 2 Stritch School of Medicine, Loyola University Chicago; 3 Division of Urology, Southern Illinois University; 4 Department of Pathology, Loyola University Medical Center

**Keywords:** leiomyosarcoma, metastasis, kidney, partial nephrectomy, desmin, smooth muscle actin

## Abstract

A 49-year-old woman with a distant history of uterine leiomyosarcoma underwent robotic-assisted laparoscopic partial nephrectomy for a 3.5 cm right renal mass, which was presumed to be a primary renal cell carcinoma. Surgical margins were negative, and the histologic analysis confirmed leiomyosarcoma. Uterine leiomyosarcoma is traditionally a locally aggressive disease with only rare reports of renal involvement. We report a case of a metastatic leiomyosarcoma to the kidney four years following initial treatment for uterine leiomyosarcoma.

## Introduction

Uterine leiomyosarcomas (LMS) are rare tumors of the uterus, which comprise roughly 1% of uterine malignancies and one-third of uterine sarcomas [1]. Uterine LMS tend to carry a poor prognosis, with a five-year survival rate of 66% [2]. For disease confined to the uterus, a total hysterectomy is the standard of care, with many surgeons also performing a bilateral salpingo-oophorectomy at the time of hysterectomy [3]. Uterine LMS do not frequently metastasize; however, when they do, metastases are most commonly found in the lungs, liver, abdomen, pelvis, and regional lymph nodes. With high recurrence rates, routine computerized tomography (CT) surveillance of patients with a history of LMS is recommended. CT scans of the chest, abdomen, and pelvis should be obtained every three to six months for the first two to three years, then every six months for the next two years, and then yearly after that [4]. 

## Case presentation

**Figure 1:**
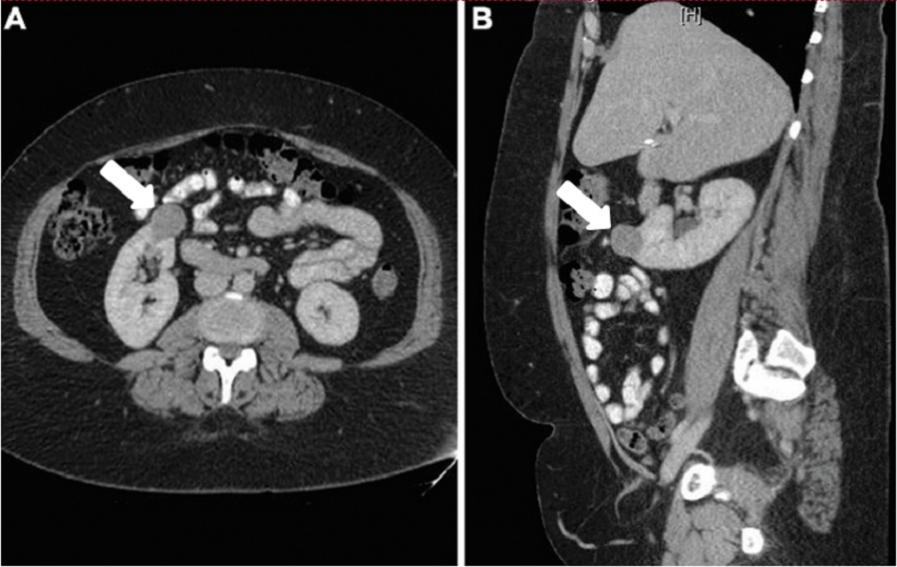
Representative contrast computed tomographic images of the renal mass A) axial and B) sagittal planes

A 49-year-old woman presented to the urology clinic with a recently diagnosed 3.5 cm right renal mass found on computed tomography (Figure 1). She was diagnosed with uterine LMS four years prior following vaginal hysterectomy for menorrhagia, for which she received adjuvant pelvic radiation. The right renal mass was noted on surveillance imaging and was new from imaging six months prior. She was seen and evaluated in the urology clinic and surgical removal of the mass was advised. Given her age and the location of the tumor, a partial nephrectomy was recommended. She subsequently underwent robotic-assisted laparoscopic right partial nephrectomy with intraoperative ultrasound guidance via a transperitoneal approach. Her postoperative course was uneventful and she was discharged home on postoperative day one.

**Figure 2:**
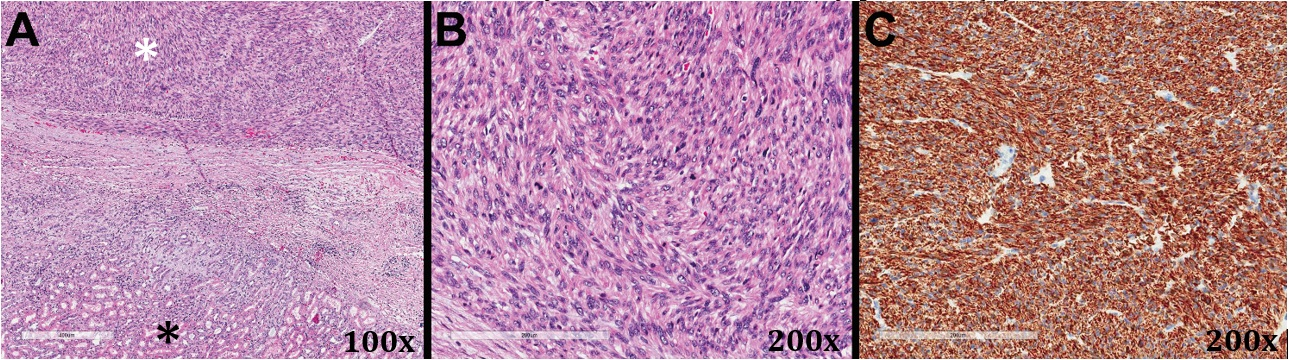
Histologic slides from the tumor specimen

Pathologic analysis by a board certified pathologist revealed negative surgical margins. On low power histologic examination, spindle-shaped cells were seen adjacent to normal renal tubules (Figure 2A, white asterisk shows spindle cells and black asterisk shows normal renal tubules). Higher magnification demonstrated high mitotic activity, characteristic of LMS (Figure 2B). Immunohistochemical stains diagnostic of LMS, desmin (Figure 2C) and smooth muscle actin (not shown), were diffusely positive. Follow-up imaging at 12 months showed no evidence of disease recurrence. She has had no other clinical evidence of disease recurrence or progression. 

## Discussion

Uterine LMS are aggressive tumors with local recurrence rates ranging from 45% to 73% [5-8]. Metastasis is uncommon but preferentially involves the lung and, less frequently, the liver and other soft tissues [9]. Only two prior reports of renal metastases exist, dating back to the 1980s [9-10]. This patient with oligometastatic disease has done well following robotic-assisted laparoscopic partial nephrectomy. Renal mass biopsy in the setting of a known, non-renal malignancy, while not universally performed, may prove useful in the setting of prior LMS where aggressive surgical resection would be indicated. This case highlights the importance of considering prior malignancies in the evaluation of a new renal mass, even if the prior malignancy is thought to be in remission.

## Conclusions

We have presented a rare case of solitary metastasis to the kidney from a uterine LMS. The patient underwent a transperitoneal robotic-assisted laparoscopic partial nephrectomy, and negative surgical margins were achieved. Thus far, three years after surgery, the patient has remained without evidence of disease. A detailed oncologic history should be obtained upon evaluation of a patient with a new renal mass to identify any potential other primary malignancies that may have caused the renal mass. Consideration should be given for preoperative biopsy for management guidance. In patients with a history of LMS or other aggressive malignancy, wider resection may be considered.
